# Comprehensive analysis of differentially expressed genes associated with PLK1 in bladder cancer

**DOI:** 10.1186/s12885-017-3884-2

**Published:** 2017-12-16

**Authors:** Zhe Zhang, Guojun Zhang, Zhipeng Gao, Shiguang Li, Zeliang Li, Jianbin Bi, Xiankui Liu, Zhenhua Li, Chuize Kong

**Affiliations:** 1 Department of Urology, First Hospital of China Medical University, 155 North Nanjing Street, Heping, Shenyang, Liaoning 110001 China; 2 Institute of Urology, China Medical University, Shenyang, 110001 China; 3 Department of Hematology, Shengjing Hospital of China Medical University, 39 Huaxiang Road, Tiexi, Shenyang, Liaoning 110022 China

**Keywords:** Bladder cancer, PLK1, Go, KEGG, BUB1B, CCNB1, CDC25A, FBXO5, NDC80

## Abstract

**Background:**

The significance of PLK1 (polo-like kinase 1) has become increasingly essential as both a biomarker and a target for cancer treatment. Here, we aimed to determine the downstream genes of PLK1 and their effects on the carcinogenesis and progression of bladder cancer.

**Methods:**

Specific siRNA was utilized to silence the target gene expression. The cell proliferation, invasion and migration of bladder cancer cells by MTT assay, BrdU assay and transwell assay. The differential expression genes were identified using Affymetrix HTA2.0 Array. The KEGG, GO and STRING analysis were used to analyze the signaling pathway and protein-protein interaction. Spearman analysis was used to analyze the correlation between protein and protein, between protein and clincopathologic characteristics.

**Results:**

PLK1 siRNA hindered the proliferation, invasion and migration of bladder cancer cells, as determined by the MTT, BrdU and transwell assays. A total of 561 differentially expressed genes were identified using an Affymetrix HTA2.0 Array in PLK1 knockdown T24 cells. According to KEGG, GO and STRING analysis, five key genes (BUB1B, CCNB1, CDC25A, FBXO5, NDC80) were determined to be involved in cell proliferation, invasion and migration. PLK1 knockdown decreased BUB1B, CCNB1, CDC25A and NDC80 expressions but increased FBXO5 expression. BUB1B, CCNB1, CDC25A and NDC80 were positively correlated with cell proliferation, invasion, migration and PLK1 expression in tissues, but FBXO5 was negatively correlated with each of those factors. The results showed that the five genes expressions were significantly correlation with the PLK1 expression in normal bladder tissues and bladder cancer tissues. Four of them (BUB1B, CCNB1, CDC25A, NDC80) were obviously positive correlations with pT stage and metastasis. But FBXO5 was negative correlated with pT stage and metastasis. Furthermore, significant correlations were found between CCNB1 or CDC25A or NDC80 and histological grade; between BUB1B or NDC80 and recurrence.

**Conclusion:**

Five downstream genes of PLK1 were associated with the regulation of cell proliferation, invasion and migration in bladder cancer. Furthermore, these genes may play important roles in bladder cancer and become important biomarkers and targets for cancer treatment.

**Electronic supplementary material:**

The online version of this article (10.1186/s12885-017-3884-2) contains supplementary material, which is available to authorized users.

## Background

Bladder cancer is a most common urological malignancy which causes approximately 150,000 deaths annually worldwide [1]. Bladder cancer is highly varied, and non-muscle-invasive bladder cancer and muscle-invasive bladder cancer are its two major subsets. Approximatedly 15–25% of non-muscle-invasive bladder cancer will progress to muscle-invasive bladder cancer [2]. Muscle-invasive bladder cancer would rapidly progress and metastasize. Though the improved therapeutic strategies are given, there is still a high mortality [3]. Appropriate risk assessment and outcome prediction are important for making better prognosis. But current staging systems may be less accurate at risk assessment. Hence, elucidating new therapeutic methods to improve its clinical prognosis is important.

Polo-like kinase 1 (PLK1) is a well-known oncogene that has well-documented roles in many cell cycle related events. PLK1 overexpression has been found in many cancer cell lines and neoplastic tissues [4–6]. Moreover, PLK1 has also been shown to play a critical role in the cell invasion and migration of many cancers [7, 8]. The PLK1 expression status was shown to be closely correlated with important histopathological characteristics of renal carcinomas and to play an important role in cell proliferation and invasion [9].

We previously determined that PLK1 plays an important role in the carcinogenesis and development of bladder cancer [10, 11]. In the current study, we aimed to clarify the mechanism underlying PLK1 knockdown-induced anticancer effects on a genome-wide level using cDNA microarray technology. The relationships between PLK1 expression and downstream target genes were also determined. The downstream genes and pathways of PLK1 in bladder cancer cells were identified by GO and KEGG enrichment analysis and a protein-protein interaction network.

## Methods

### Clinical samples

A collection of 50 bladder cancer samples were obtained from patients who underwent partial cystectomy or radical cystectomy from 2012 to 2016 at the Department of Urology of the First Hospital of China Medical University in China. 20 normal bladder epithelial tissues were from patients with benign prostatic enlargement. The study was conducted according to a protocol approved by an institutional review board (2017–37) of the Medical Ethics Committee of the First Hospital of China Medical University, and written informed consent was obtained from each patient for surgical and research purposes. Histologically, tumors were classified according to the 2004 World Health Organization histological classification of urinary tract tumors [12]; 29 papillary urothelial carcinomas and 21 invasive urothelial carcinomas were included in the study. The tumors were staged using the 2002 TNM classification [13]; 22 Lower stage bladder carcinomas (Ta) and 28 higher stage bladder carcinomas (≥pT1) were included. None of the cancer patients received adjuvant chemotherapy or radiation therapy before surgery. All patients with bladder reservation received routine urine examinations, chest X-rays, abdominal and pelvic ultrasonography examinations, cystoscopies, and cytology examinations every 3 months. During the follow-up period, tumor metastasis (local lymph node metastasis) and recurrence (pathologically proven locoregional recurrence) were observed in 10 and 19 patients, respectively. The study was carried out with human tissue samples as well as cell lines.

### Cell culture and transfection

The normal bladder epithelial cell line SV-HUC-1 (SV-40 immortalized human uroepithelial cell line) and the bladder cancer cell lines RT4, BIU-87, 5637 and T24 were obtained from the Chinese Academy of Sciences Cell Bank (CASCB, China). The cells were cultured in RPMI 1640 medium (Gibco, USA) supplemented with 10% heat-inactivated fetal bovine serum (FBS) (Gibco, USA) at 37 °C in 5% CO_2_.

Cells were transfected with double-stranded siRNA oligomers using Lipofectamin^®^ 3000 tranfection reagent (Life Technologies Corporation, USA) according to the manufacturer’s instructions. Briefly, cells were seeded into 6-well plates at a density of 1 × 10^6^ cells per well and grown for 12 h prior to transfection with specific siRNA of the target genes for 48 h. The specific siRNA of the target genes and the control negative siRNA were purchased from GenePharma (GenePharma Corporation, China) and listed in Additional file 1: Table S1.

### Quantitative real-time polymerase chain reaction

Total RNA was extracted from tissues or cultured cells with TRIzol reagent (Invitrogen, Carlsbad, CA) according to the manufacturer’s instruction. RNA was reverse transcribed into first-strand cDNA using PrimeScript™ RT Master Mix (Perfect Real Time; Takara Biotechnology Co. Ltd., Dalian, China) according to the manufacturer’s instructions. Real-time qPCR was carried out to detect the levels of the corresponding GAPDH, PLK1, BUB1B, CCNB1, CDC25A, FBXO5 and NDC80 genes using SYBR® Premix Ex Taq™ (Tli RNaseH Plus; Takara Biotechnology Co. Ltd., Dalian, China) and a Thermal Cycler Dice™ Real Time TP800 system (Takara, Kyoto, Japan). GAPDH was used as an internal control for each specific gene. The reaction was heated to 55 °C for 2 min, 95 °C for 10 min by 35 cycles, denaturation at 95 °C for 15 s, annealing at 60 °C for 30 s, and extension at 72 °C for 30 s. The primer sequences for the target genes are shown in Additional file 2: Table S2. The relative expression levels were quantified and analyzed using SDS 2.3 software (Applied Biosystems, NY, USA). The real-time value for each sample was averaged and compared using the Ct method. The relative expression levels (defined as fold change) of the target genes (2-ΔΔCt) were normalized to the endogenous GAPDH reference (ΔCt) and related to the amount of target gene in the control sample, which was defined as the calibrator at 1.0. Three independent experiments were carried out to analyze relative gene expression, and each sample was tested in triplicate.

### Western blotting

Total protein was extracted using Pierce lysis buffer (Pierce, Rockford, IL). Protein quantification was performed using the Bradford method (Bio-Rad Co., USA). Proteins were separated using sodium dodecyl sulfate polyacrylamide gel electrophoresis (SDS-PAGE) and transferred to polyvinylidene fluoride (PVDF) membranes. The membranes were blocked in Tris buffered saline tween (TBST) with low-fat milk and then incubated overnight with primary antibodies against PLK1 (1:1000, ab109777, Abcam, USA), BUB1B (1:1000, ab70544, Abcam, CA, USA), CCNB1 (1:1000, ab2949, Abcam, CA, USA), CDC25A (1:1000, ab989, Abcam, CA, USA), FBXO5 (1:1000, ab129905, Abcam, CA, USA), NDC50 (1:3000, SAB1410085, Sigma, CA, USA) and GAPDH (1:2000, ab9485, Abcam, CA, USA) at 4 °C. The membranes were then washed with TBST and incubated with the horseradish peroxidase-conjugated secondary antibody goat anti-rabbit IgG (1:5000, Sigma, CA, USA). The blots were developed with ECL solution (Pierce, Rockford, IL, USA) and detected using a chemiluminescence system (Bio-Rad, CA, USA). Image Lab software was employed to analyze the intensities of the band signals obtained.

### 3-(4,5-dimethylthazol-2-yl)-2,5-diphenyltetrazolium bromide (MTT) assay

Approximately 5000 cells were seeded into 96-well culture plates. After the cells had adhered, the intervention factor corresponding to each category was applied to each group in three repeated wells. After culture, cell growth was measured following the addition of a 0.5 mg/ml MTT (Sigma-Aldrich, USA) solution. Approximately 4 h later, the medium was replaced with 100 ml of DMSO (Sigma-Aldrich, USA) and vortexed for 10 min. Absorbance was measured at a wavelength of 490 nm using a plate reader (model 680, Bio-Rad, Hertfordshire, UK).

### BrdU incorporation assay

In total, 1 × 10^5^ cells were seeded into 24-well culture plates. After the cells had adhered, the intervention factor corresponding to each category was applied to each group in three repeated wells. Cells were then fixed in paraformaldehyde for 20 min and 0.1% Triton X-100 for 5 min. The cells were washed with PBS and then blocked with 3% BSA for 1 h at 37 °C. Anti-BrdU diluted in 3% BSA was added overnight. The cells were washed 3 times with PBS and then incubated with a TRITC-labelled goat anti-mouse antibody for 1 h at room temperature. They were then washed with PBS 3 times, and nuclei were stained with DAPI (1 μg/ml) for 1 min. The cells were once again washed with PBS before being observed and captured on a fluorescence microscope (×100, Olympus).

### Transwell migration and invasion assay

Cell migration and invasion were determined using a transwell chamber (8 μm pore size) with and without BD Matrigel (BD Biosciences, CA, USA). The upper side of the membrane was coated with Matrigel for the invasion assay. After 48 h of transfection, 1 × 10^5^ cells were added to the upper chamber, medium (500 μL) containing 10% FBS was added to the lower chamber, and the apparatus was incubated at 5% CO_2_ and 37 °C. The membranes were fixed at 24 h and stained with 0.5% crystal violet (Sigma, USA). After removing the non-motile cells at the tops of the membranes with cotton swabs, 5 visual fields of each membrane were randomly selected and counted at 200× magnification.

### Microarray-based gene expression profiling and data analysis

Gene expression profiling analysis was performed by Shanghai Biotechnology Corporation (Shanghai, China). For total RNA isolation, the RNAeasy Mini Kit (Qiagen, CA, USA) was used according to the manufacturer’s protocol. RNA quantity and purity were determined by optical density measurements (OD260/OD280), and RNA integrity was assessed using the NanoDrop 2000 spectrophotometer (Thermo Scientific, DE, USA). For Affymetrix HTA 2.0 array analysis, 500 ng of RNA extracted from PLK1 knockdown and T24 control cells (three independent samples each) was processed to generate biotinylated hybridization targets using One Cycle cDNA Synthesis and One Cycle Target Labelling Kits from Affymetrix (Affymetrix, CA, USA) according to the manufacturer’s protocols. Labeled cDNAs were fragmented and hybridized against the GeneChip arrays. The arrays were scanned using a Hewlett Packard confocal laser scanner and analyzed with MicroArray Suite 5.0 software (Affymetrix, CA, USA). The functions and related pathways of the differentially expressed genes were further analyzed using the Gene Ontology (GO) and Kyoto Encyclopedia of Genes and Genomes (KEGG) databases. The protein-protein networks of the identified expression genes were mapped using STRING software to predict protein interactions. By integrating these correlations, interaction networks between the target genes and their interactive genes were constructed.

### Statistical analysis

Statistical analysis was performed using SPSS (Statistical Package for the Social Sciences) 17.0 (SPSS Inc., Chicago, IL). The results are presented as the mean ± SD unless otherwise stated. *P* < 0.05 was considered to indicate significant differences of two-tailed test. Multiple samples were compared using analysis of Variance Analysis. Two-two comparisons among multiple variables were analyzed using Turkey’s multiple comparisons test. Two-two comparisons between two independent variables were analyzed using Student’s T test. Correlations between two variables were analyzed using Spearman rank correlation analysis.

## Results

### PLK1expression in bladder cancer cell lines

**Fig. 1:**
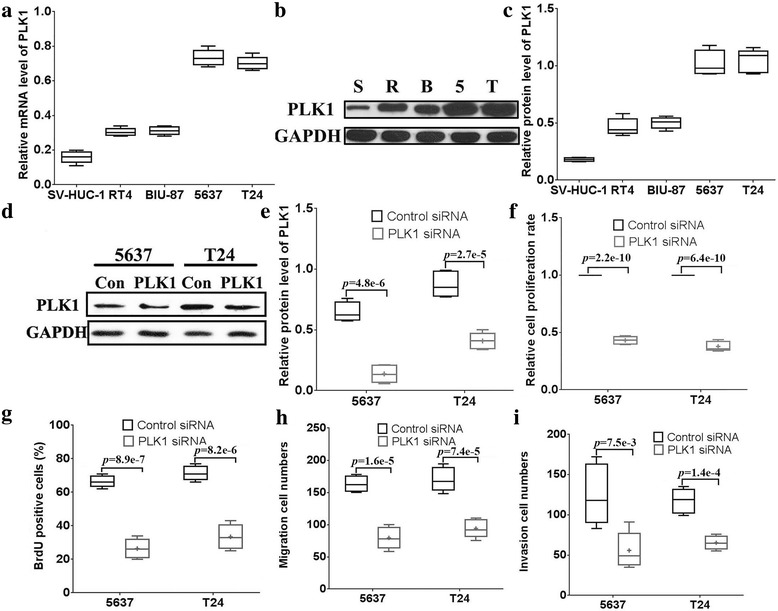
PLK1 knock-down hinders cell proliferation, invasion and migration. The mRNA (**a**) and protein expression (**b, c**) levels of PLK1 were examined by qPCR and western blotting. S: SV-HUC-1, R: RT4, B: BIU-87, 5: 5637, T: T24. The efficiency of PLK1 knockdown by siRNA was determined by western blotting (**d, e**). The cell proliferation was examined by the MTT assay (**f**) and BrdU assays (**g**) in control siRNA groups and the PLK1 siRNA group. The transwell assay was used to examine the cell migration (**h**) and (**i**) invasion in control siRNA group and PLK1 siRNA groups

To investigate the potential role of PLK1 in bladder cancer, the mRNA and protein expression levels of PLK1 were examined in RT4, BIU-87, 5637 and T24 cells and the SV-HUC-1 cells using real-time PCR and western blotting. As shown in Fig. 1a-c, both the PLK1 mRNA and protein expression levels were remarkably higher in RT4, BIU-87, 5637, T24 cells than that in SV-HUC-1 cells. Furthermore, the PLK1 expression levels in 5637 and T24 cells were significantly higher than those in RT4 and BIU-87 cells. Hence, 5637 and T24 cells were utilized in the subsequent PLK1 silencing experiments.

### PLK1 knock-down hinders cell proliferation, invasion and migration

To explore the function of PLK1 in bladder cancer cells, PLK1-specific siRNA was used to silence its expression (Fig. 1d-e). Cell proliferation ability was examined by the MTT assay, and 5637 and T24 cells transfected with PLK1 siRNA grew slower than those transfected with control siRNA (Fig. 1f). Moreover, the BrdU cell proliferation assay showed that cell proliferation rates in PLK1 siRNA-treated 5637 and T24 cells were decreased compared to those in control cells (Fig. 1g). Together, these results reveal that reduced PLK1 expression may attenuate the proliferation ability of bladder cancer cells.

Next, the functions of PLK1 in regulating cell invasion and migration were also detected in 5637 and T24 cells by the transwell migration assay. The invasion cell numbers of 5637 and T24 cells treated with PLK1 siRNA were 49 ± 14 and 65 ± 11, respectively (Fig. 1h), which were lower than those of the control groups (98 ± 20 and 119 ± 16, respectively). Furthermore, the migratory cell numbers of 5637 and T24 cells treated with PLK1 siRNA were 78 ± 21 and 91 ± 19, respectively (Fig. 1i), which were lower than those of the control groups (162 ± 15 and 167 ± 26, respectively). These results demonstrated that PLK1 may play an important role in the invasion and migration of bladder cancer cells in vitro.

### Gene expression microarray analyses of PLK1 target genes in bladder cancer cells

**Fig. 2:**
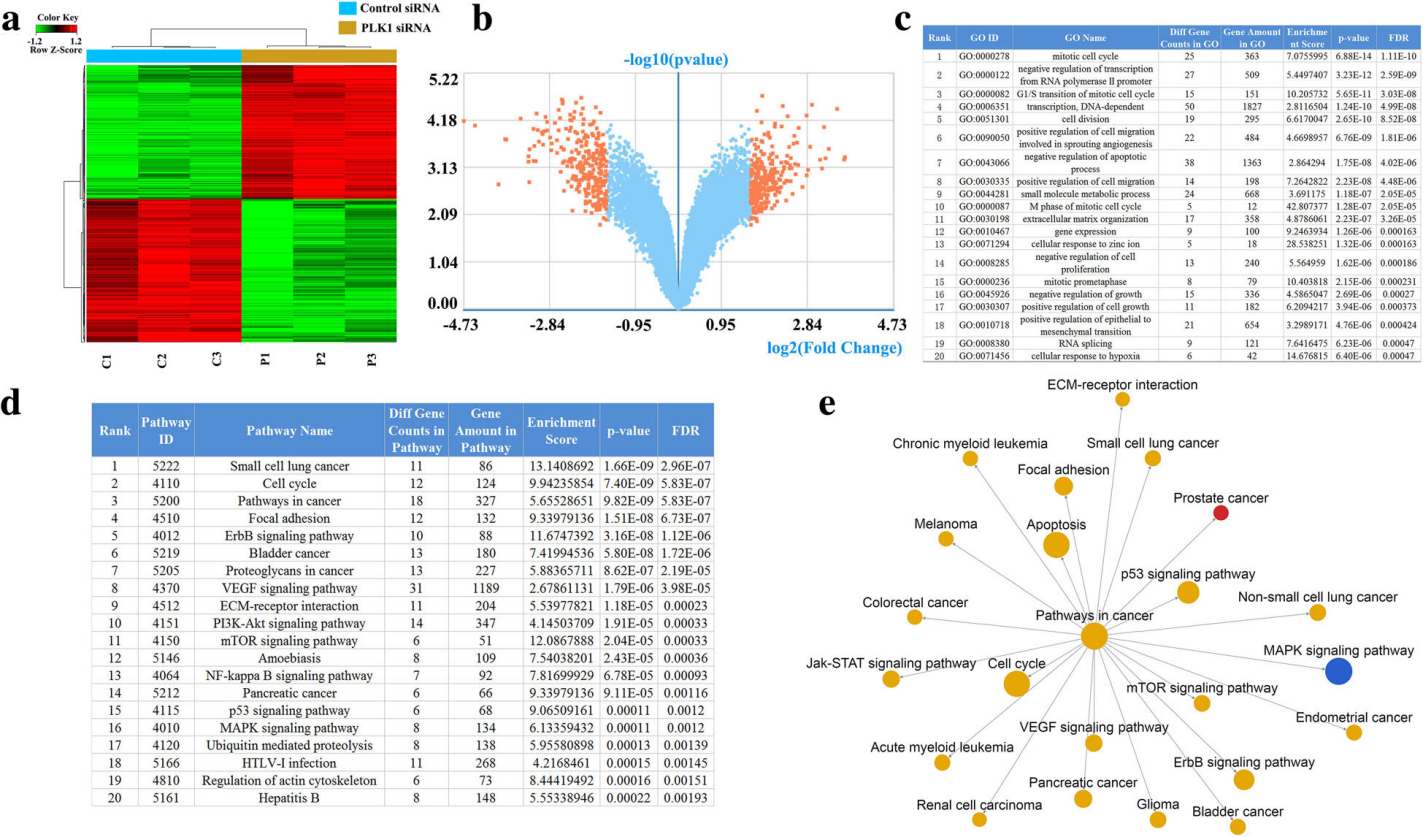
Gene expression microarray analyses of the target genes of PLK1 target genes in bladder cancer cells. The A heatmap (**a**) and volcano map (**b**) showed showing the differentially expression expressed genes analyzed by the Affymetrix HTA 2.0 Array in control siRNA group and PLK1 siRNA groups. C: control siRNA, P: PLK1 siRNA. The A summary of the top 20 changaltered biological processes or pathways after knockdown of PLK1 knockdown in T24 cells by using GO biological process analysis (**c**) and KEGG pathway analysis (**d**). Network analysis of the pathways after knockdown of PLK1 knockdown in T24 cells (**e**)

To investigate the molecular mechanisms underlying how PLK1 regulates the functions of bladder cancer cells, gene expression microarray was performed to examine differentially expressed genes after PLK1 inhibition. In total, 561 genes were identified as being significantly changed (Q < 0.05, *P* < 0.05, fold change > 3) after PLK1 knockdown in T24 cells (Fig. 2a-b). According to KEGG (Kyoto Encyclopedia of Genes and Genomes) pathway and GO (Gene Ontology) analysis regarding the target genes of PLK1, obviously enriched functions and signaling pathways were associated with PLK1 knockdown. A total of 136 enriched GO terms and 69 KEGG pathways were obtained. GO biological process analysis showed that genes from the top 20 enriched GO terms mainly participate in the mitotic cell cycle, cell proliferation and cell migration (Fig. 2c). KEGG pathway analysis also indicated that genes from the top 20 enriched KEGG pathways were significantly involved in the cell cycle, cell proliferation, cell adhesion and EMC (Fig. 2d). Moreover, network analysis of these important pathways was performed. These pathways were correlated with the cancer process (Fig. 2e). Additionally, correlations between PLK1 and cellular proliferation, migration and invasion processes existed.

### Key downstream genes were identified in PLK1 signaling pathway

**Fig. 3:**
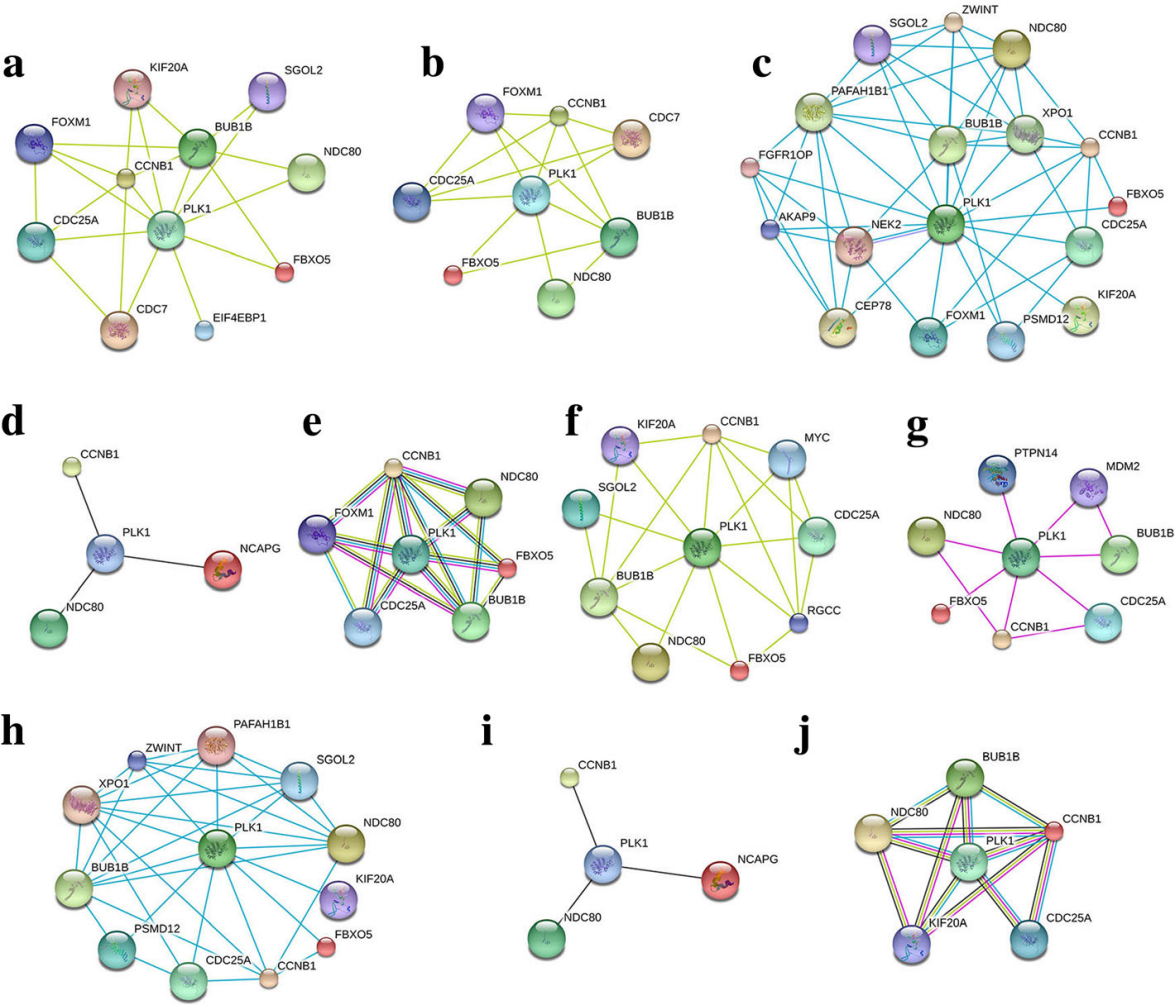
Key downstream genes were identified in the PLK1 signaling pathway. Protein-protein interaction analysis was used to screen the important candidate genes regulated by PLK1 in bladder cancer cells by using STRING software (http://string-db.org). Analysis of the interaction between PLK1 and the differentially expressed genes about associated with cell proliferation signaling pathways by textmining (**a**), Experiments experiments (**b**), Database database (**c**) and, Coco-expression (**d**) and multiple methods (**e**). Analysis of the interaction between PLK1 and differentially expressed genes about associated with cell invasion and migration signaling pathways by textmining (**f**), Experiments experiments (**g**), Database database (**h**) and, Coco-expression (**i**) and multiple methods (**j**)

PLK1 was determined to be involved in the regulation of cell proliferation, invasion and migration by some signaling pathways. To determine the key genes regulated by PLK1 in bladder cancer cells, we further analyzed the significantly altered genes related to cell proliferation, invasion and migration. We performed protein-protein interaction analysis to screen important candidate genes regulated by PLK1 in bladder cancer cells using STRING software (http://string-db.org). In total, 69 differentially expressed genes associated with cell proliferation signaling pathways were identified from GO and KEGG pathway analyses. Four protein-protein interaction analysis methods were used with STRING software: textmining, experimental, database and co-expression. Using the four methods, 10, 7, 16 and 3 differentially expressed genes were determined to be regulated by PLK1, respectively (Fig. 3a-d, Additional file 3: Table S3). Among them, 6 key genes (*BUB1B, CCNB1, CDC25A, FBXO5, FOXM1, NDC80*) were closely correlated with PLK1 (Fig. 3e).

Moreover, 70 differentially expressed genes associated with cell adhesion, migration and EMC signaling pathways identified from GO and KEGG pathway analyses were selected. Through textmining, experimental, database, and co-expression analysis, 9, 7, 11 and 3 differential genes were determined to be regulated by PLK1, respectively (Fig. 3f-i, Additional file 4: Table S4). Among them, 6 key genes (*BUB1B, CCNB1, CDC25A, FBXO5, KIF20A, NDC80*) were closely correlated with PLK1 (Fig. 3j). Compared with the key genes regulated by PLK1 in the cell proliferation process, five of the same genes (*BUB1B, CCNB1, CDC25A, FBXO5, NDC80*) were also determined to be involved in the PLK1 pathway associated with cell invasion and migration.

### Validation of the five representative key genes regulated by PLK1 in bladder cancer cells

**Fig. 4:**
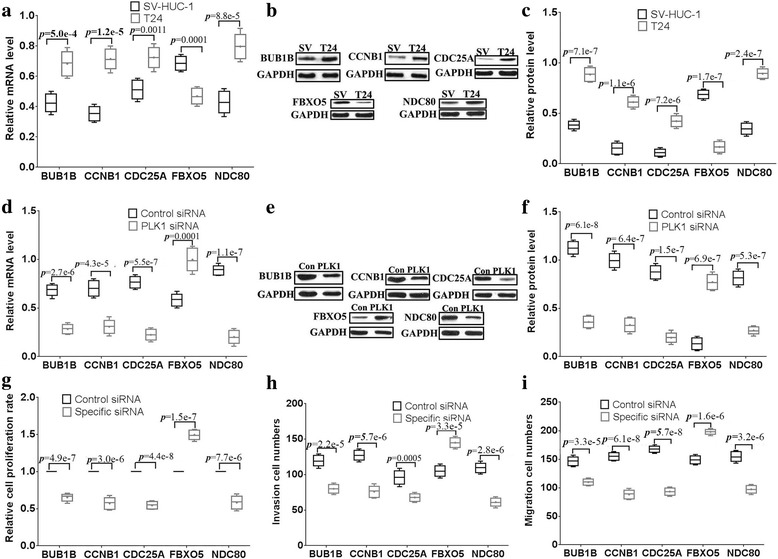
Validation ofe the five representative key genes regulated by PLK1 in bladder cancer cells. The mRNA (**a**) and protein expression (**b, c**) levels of five genes (BUB1B, CCNB1, CDC25A, FBXO5, NDC80) were examined by qPCR and western blotting. SV: SV-HUC-1, T24: T24. The mRNA (**d**) and protein expression (**e, f**) levels of the five genes were determined in T24 cells with PLK1 knockdown by qPCR and western blotting. The Cell proliferations were abilities were examined by the MTT assay (**g**) in the five genes specific siRNA groups. The transwell assay was used to examine the cell migration (**h**) and (**i**) invasion in the five genes gene-specific siRNA groups

First, the mRNA and protein expression levels of the five key genes (*BUB1B, CCNB1, CDC25A, FBXO5, NDC80*) were examined in SV-HUC-1 and T24 cells. BUB1B, CCNB1, CDC25A and NDC80 were expressed at higher mRNA and protein levels in T24 cells than in SV-HUC-1 cells, but FBXO5 was expressed at lower mRNA and protein levels in T24 cells than in SV-HUC-1 cells (Fig. 4a-c). When PLK1 siRNA was applied to T24 cells, the mRNA and protein expression levels of BUB1B, CCNB1, CDC25A and NDC80 were significantly decreased, while the mRNA and protein expression levels of FBXO5 were increased (Fig. 4d-f). Furthermore, siRNAs specific to the five key genes were applied to T24 cells. Cellular proliferation, invasion and migration abilities were hindered in the siRNA-specific (BUB1B, CCNB1, CDC25A and NDC80) groups compared with those in control siRNA groups, but FBXO5 siRNA promoted cell proliferation, invasion and migration (Fig. 4g-i). These results suggested that the five key genes are regulated by PLK1 and are involved in PLK1 signaling pathways in the regulation of the proliferation, invasion and migration of bladder cancer cells.

### Analysis of the correlation between the five key genes and PLK1 in bladder cancer tissues

**Fig. 5:**
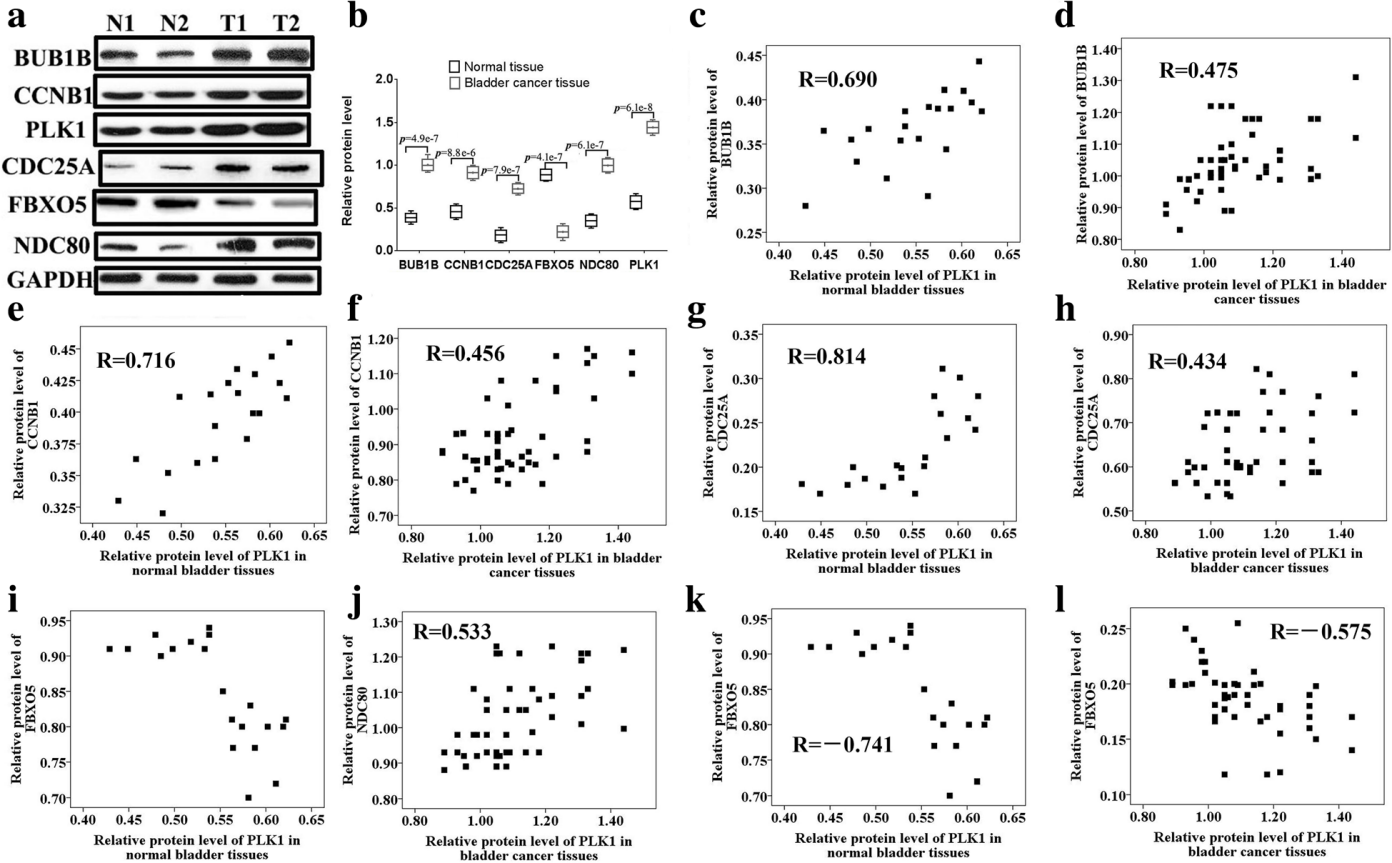
Analysis of the cCorrelation analysis between the five key genes and PLK1 in bladder cancer tissues. The protein expression (**a**, **b**) levels of the five genes (BUB1B, CCNB1, CDC25A, FBXO5, NDC80) were examined by western blotting. Spearman correlation analysis was applied to compare the relative protein expression levels of PLK1 and the single genes in normal bladder tissues (**c**, **e**, **g**, **i**, **k**) and bladder cancer tissues (**d**, **f**, **h**, **j**, **l**)

To determine the relationship between PLK1 and the five genes, we examined the protein expression levels of PLK1 and the five genes in 50 bladder cancer tissues and 20 normal bladder epithelial tissues by western blotting. Four genes (BUB1B, CCNB1, CDC25A and NDC80) were expressed at higher levels in bladder cancer tissues than in normal bladder tissues, but the expression of FBXO5 was lower in bladder cancer than in normal tissues (Fig. 5a-b, Additional file 5: Table S5 in Additional files). Furthermore, Spearman correlation analysis was applied to compare the relative protein expression levels of PLK1 and the single genes in these bladder tissues. The protein expression levels of PLK1 were positively correlated with those of BUB1B (Fig. 5c (normal bladder tissues), *R* = 0.690, *p* < 0.01; Fig. 5d (bladder carcinomas), *R* = 0.475, *p* < 0.01), CCNB1 (Fig. 5e (normal bladder tissues), *R* = 0.716, *p* < 0.01; Fig. 5f (bladder carcinomas), *R* = 0.456, *p* < 0.01), CDC25A (Fig. 5g (normal bladder tissues), *R* = 0.814, *p* < 0.01; Fig. 5h (bladder carcinomas), *R* = 0.434, *p* < 0.01) and NDC80 (Fig. 5i (normal bladder tissues), *R* = 0.760, *p* < 0.01; Fig. 5j (bladder carcinomas), *R* = 0.533, *p* < 0.01) but negatively correlated with those of FBXO5 (Fig. 5k (normal bladder tissues), *R* = −0.741, *p* < 0.01; Fig. 5l (bladder carcinomas), *R* = −0.575, *p* < 0.01). The results illustrated that the expression levels of the five genes were significantly correlated with PLK1 expression in normal bladder tissues and bladder cancer tissues.

### Association of the protein expression of the five key genes with the clinicopathological characteristics of bladder cancer patients

**Fig. 6:**
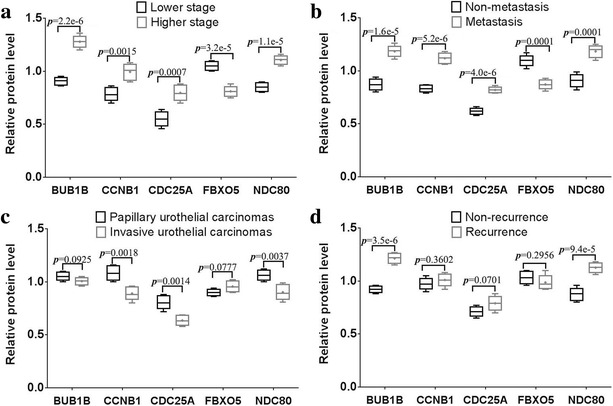
Association of the protein expression of thes five key genes with the clinicopathological characteristics of the bladder cancer patients. Western blotting was used to determine the relationship between the expression of the five proteins (BUB1B, CCNB1, CDC25A, FBXO5, NDC80) expression and clinicopathological features (stage (**a**), grade (**b**), metastasis (**c**) and recurrence (**d**))

To evaluate the significance of the five proteins in bladder cancer, we investigated the relationship between the expression of the five proteins (BUB1B, CCNB1, CDC25A, FBXO5, NDC80) and clinicopathological features. Overall, four proteins (BUB1B, CCNB1, CDC25A, NDC80) were obviously positively correlated with pT stage (Fig. 6a) and metastasis (Fig. 6b). However, FBXO5 was negatively correlated with pT stage (Fig. 6a) and metastasis (Fig. 6b). Furthermore, significant correlations were found between CCNB1, CDC25A and NDC80 and histological grade (Fig. 6c) and between BUB1B and NDC80 and recurrence (Fig. 6d). Therefore, the five proteins (BUB1B, CCNB1, CDC25A, FBXO5, NDC80) are closely correlated with important clinicopathological characteristics (stage, grade, metastasis and recurrence).

## Discussion

Bladder carcinoma has become the most frequent neoplasm of the urinary tract, involving distinct and multiple molecular pathologies. While several of these changes have been described, many more are being detected. When additional molecular determining factors are added to a continuously increasing list of prognostic indicators for bladder cancer, the need to integrate these markers into logical groups and use them to confirm cancer progression and prognosis increases.

Increasing evidence supports that PLK1 has multiple non-mitotic functions, especially in cancer cells. In previous experiments, we revealed that PLK1 is upregulated in bladder cancer tissues and is thus associated with malignancy [10, 11]. In this study, we found that the proliferation, invasion and migration of bladder cancer cells decreased upon PLK1 knockdown. Whole-gene expression microarray analysis of PLK1 knockdown in T24 cells identified 561 differentially expressed genes. KEGG and GO analysis then suggested that PLK1 mainly modulates genes related to the cell cycle and cell migration and invasion in bladder cancer. We performed protein-protein interaction analysis to select five important candidate genes (BUB1B, CCNB1, CDC25A, FBXO5, KIF20A, NDC80) regulated by PLK1 in bladder cancer cells using STRING software. The subsequent research focused on the relationship between these five genes and PLK1 and their functions in bladder cancer.

BUB1B, a mitotic checkpoint protein, is a key component of the mitotic spindle checkpoint complex [14]. Moreover, some studies have proven the role of BUB1B in cancers. BUB1B may contribute to gastric tumorigenesis and the risk of tumor development [15]. Overexpression of BUB1B in prostate cancer cells promotes cell proliferation and migration [16]. BUB1B was expressed higher in invading metastasized breast cancer cells than in those without metastasis [17]. BUB1B localizes to centrosomes, physically interacts with PLK1 and inhibits the phosphorylation and kinase activity of PLK1 during interphase [18]. In our study, we determined a positive correlation between PLK1 and BUB1B both in vivo and in vitro. Furthermore, BUB1B was closely correlated with important clinicopathological characteristics (stage, metastasis and recurrence).

Both CDC25A and CCNB1 are cell cycle-related proteins. CDC25A, a dual-specificity phosphatase, removed inhibitory phosphorylation in cyclin-dependent kinases (CDKs) and positively regulated the activities of CDKs [19]. In HEK-293 cells, CDC25A inhibited cisplatin-induced apoptotic cell death by stimulating nuclear factor-kappa B activity [20]. CDC25A expression showed significant correlation with poor tumor differentiation and tumor invasion in retinoblastoma [21]. Tumor CDC25A expression was strongly associated with metastatic diseases in hepatocellular carcinoma, and PLK1 could be an upstream regulator of CDC25A [22]. The degradation of CCNB1 by PLK1 inhibition appeared to be a critical promoter of mitotic slippage [23]. However, in head-and-neck squamous cell carcinoma, PLK1 siRNA significantly increased the CCNB1 mRNA level [24]. Our data showed a positive correlation between PLK1 and both CDC25B and CCNB1. Furthermore, both CDC25B and CCNB1 were closely correlated with important clinicopathological characteristics (stage, grade and metastasis).

FBXO5 (also known as EMI1) inhibited the anaphase-promoting complex, which controls cell cycle progression through the sequential degradation of various substrates [25]. FBXO5 was degraded during the mitosis prophase via a PLK1-dependent pathway [26, 27]. PLK1 phosphorylated FBXO5 to ensure mitosis entry [28]. In our study, a negative correlation was confirmed between PLK1 and FBXO5. Furthermore, FBXO5 was negatively correlated with clinical stages and metastasis.

NDC80, a kinetochore outer layer component and spindle checkpoint regulator, is highly expressed in a variety of human cancers [29]. NDC80 promoted the proliferation and metastasis of colon cancer cells [30]. NDC80 overexpression was correlated with the prognosis of pancreatic cancer and regulated cell proliferation [31]. Inhibition of PLK1 expression by siRNA halted the normal kinetochore association of NDC80 and other factors [32]. Our results displayed a positive correlation between PLK1 and NDC80. Furthermore, NDC80 was closely correlated with important clinicopathological characteristics (stage, grade, recurrence and metastasis).

Above all, our results showed that efficient siRNA-mediated PLK1 knockdown might inhibit the proliferation, invasion and migration of bladder cancer cells. Microarray analysis indicated that PLK1 knockdown led to the upregulation or downregulation of downstream target genes. Bioinformatics analysis showed a correlation between PLK1 and cellular proliferation, migration and invasion processes. Meanwhile, five key genes were identified as being associated with PLK1 (BUB1B, CCNB1, CDC25A, FBXO5, NDC80). BUB1B, CCNB1, CDC25A and NDC80 were positively regulated by PLK1, and the positive correlation was associated with important clinicopathological characteristics. siRNAs specific to each of the genes inhibited bladder cancer cell proliferation, invasion and migration. However, FBXO5 was negatively regulated by PLK1, which was associated with important clinicopathological characteristics, and FBXO5 siRNA promoted bladder cancer cell proliferation, invasion and migration. These results provide a direction for additional studies. In the future, we will continue to clarify the molecular mechanism underlying the interaction between PLK1 and the five key genes and determine the mechanism and clinical significance of the five key genes in bladder cancer, which will aid the clinical diagnosis and treatment of bladder cancer.

## Conclusion

These results provide a direction for additional studies. In the future, we will continue to clarify the molecular mechanism underlying the interaction between PLK1 and the five key genes and determine the mechanism and clinical significance of the five key genes in bladder cancer, which will aid the clinical diagnosis and treatment of bladder cancer.

## Additional files

Additional file 1: Table S1. The siRNA sequences of the target genes. (DOCX 15 kb)

Additional file 2: Table S2. The primer sequences of the target genes. (DOCX 15 kb)

Additional file 3: Table S3. Analysis of cell cycle related genes regulated by PLK1 in T24 cells by the STRING software (http://string-db.org). (DOCX 15 kb)

Additional file 4: Table S4. Analysis of cell invasion and migration related genes regulated by PLK1 in T24 cells by the STRING software (http://string-db.org). (DOCX 15 kb)

Additional file 5: Table S5. Turkey’s multiple comparisons test was used in Fig. 1a-c. (DOC 56 kb)

## Abbreviations

BUB1B: Budding Uninhibited By Benzimidazoles 1
CCNB1: Cyclin B1
CDC25A: Cell Division Cycle 25A
DAPI: 4′,6-diamidino-2-phenylindole
DMSO: Dimethylsulfoxyde
ERK: Mitogen-Activated Protein Kinase
FBXO5: F-Box Protein 5
GO: Gene Ontology
KEGG: Kyoto Encyclopedia of Genes and Genomes
MTT: 3-(4,5-dimethylthazol-2-yl)-2,5- diphenyltetrazolium bromide
PI3K: Phosphatidylinositol-4,5-Bisphosphate 3-Kinase
PLK1: Polo-like kinase 1
PVDF: Polyvinylidene fluoride
SDS-PAGE: Sodium dodecyl sulfate polyacrylamide gel electrophoresis
siRNA: Small interfering RNA
TBST: Tris Buffered Saline Tween
