# Supplementary material for: Comprehensive analysis of differentially expressed genes associated with PLK1 in bladder cancer
Source: BMC Cancer. 2017 Dec 16;17:861. doi: 10.1186/s12885-017-3884-2 (PMC5732388; doi:10.1186/s12885-017-3884-2)
Supplement: Supplementary file 1 — The siRNA sequences of the target genes. (DOCX 15 kb) [file 12885_2017_3884_MOESM1_ESM.docx]

Table S1. The siRNA sequences of the target genes.

| **Target gene** | **sequence** |
| --- | --- |
| Control siRNA-1 | GGCCTATGACGGGCGGACCGCATTT |
| PLK1 siRNA | GGCCAATCAGTGGCGCGCAGGCTTT |
| Control siRNA-2 | GACCGTGGAGAAGAACGAGAACGAA |
| BUB1B siRNA | GACCAGTGCAGAGAAGAGAGCAGAA |
| Control siRNA-3 | GGTTGACTATAGAGTCGTATAGGTA |
| CCNB1 siRNA | GGTGAGTCAATATAGTGGCATTGTA |
| Control siRNA-4 | CAGTGGGTAAGAGGTGTAGGCCTCG |
| CDC25A siRNA | CAGCCTGGGTAAGAGGTGTAGGTCG |
| Control siRNA-5 | AAATCACTACAAATAACAACTTAAT |
| FBXO5 siRNA | AAATTCTCAATACAAATCAAACAAT |
| Control siRNA-6 | GCGTTGGGAGGGTCCTGCGAGCGTC |
| NDC80 siRNA | GCGCGGGTTGGAGCCTGGCGTAGTC |
