# Supplementary material for: Comprehensive analysis of differentially expressed genes associated with PLK1 in bladder cancer
Source: BMC Cancer. 2017 Dec 16;17:861. doi: 10.1186/s12885-017-3884-2 (PMC5732388; doi:10.1186/s12885-017-3884-2)
Supplement: Supplementary file 2 — The primer sequences of the target genes. (DOCX 15 kb) [file 12885_2017_3884_MOESM2_ESM.docx]

Table S2. The primer sequences of the target genes.

| **Target gene** | **Forward primer sequence** | **Reverse primer sequence** |
| --- | --- | --- |
| PLK1 | CAAGAAGAATGAATACAGTA | GGATATAGCCAGAAGTAA |
| BUB1B | CATTATCACAGGCTTCAG | CTTCAAGGACATTATCTCAT |
| CCNB1 | AATACCTGATGGAACTAA | TAACTGGAAGAAGAGATT |
| CDC25A | AATCCTATGAGAAGAATACA | GGTCAAGAGAATCAGAAT |
| FBXO5 | ACCAAGTTATCCAATCAA | GAATTACAGCGAATACAG |
| NDC80 | AATATCATTGACAACCAGAAG | CTCCTCATTCCACAACTT |
