# Supplementary material for: Comprehensive analysis of differentially expressed genes associated with PLK1 in bladder cancer
Source: BMC Cancer. 2017 Dec 16;17:861. doi: 10.1186/s12885-017-3884-2 (PMC5732388; doi:10.1186/s12885-017-3884-2)
Supplement: Supplementary file 4 — Analysis of cell invasion and migration related genes regulated by PLK1 in T24 cells by the STRING software (http://string-db.org). (DOCX 15 kb) [file 12885_2017_3884_MOESM4_ESM.docx]

Table S4. Analysis of cell invasion and migration related genes regulated by PLK1 in T24 cells by the STRING software (http://string-db.org).

| **Interaction source** | **Different gene Symbols** | **Different gene counts** |
| --- | --- | --- |
| Textmining | BUB1B,CCNB1,CDC25A, FBXO5, KIF20A,MYC,NDC80,RGCC,SGOL2 | 9 |
| Experiments | BUB1B,CCNB1,CDC25A, FBXO5,MDM2, NDC80,PTPN14 | 7 |
| Database | BUB1B,CCNB1,CDC25A, FBXO5, KIF20A,NDC80, PAFAH1B1,PSMD12,SGOL2,XPO1,ZWINT | 11 |
| Co-expression | CCNB1,NCAPG,NDC80 | 3 |
| multi-methods | BUB1B,CCNB1,CDC25A,FBX05,KIF20A,NDC80 | 6 |
