# Supplementary material for: Comprehensive analysis of differentially expressed genes associated with PLK1 in bladder cancer
Source: BMC Cancer. 2017 Dec 16;17:861. doi: 10.1186/s12885-017-3884-2 (PMC5732388; doi:10.1186/s12885-017-3884-2)
Supplement: Supplementary file 5 — Turkey’s multiple comparisons test was used in Fig. 1a-c. (DOC 56 kb) [file 12885_2017_3884_MOESM5_ESM.doc]

Table S5. Turkey’s multiple comparisons test was used in Fig1a and 1c.

| Tukey's multiple comparisons test | Mean Diff. | 95% CI of diff. | Significant |
| --- | --- | --- | --- |
|  |  |  |  |
| SV-HUC-1 vs. RT4 | -0.1440 | -0.2079 to -0.08010 | Yes |
| SV-HUC-1 vs. BIU-87 | -0.1520 | -0.2159 to -0.08810 | Yes |
| SV-HUC-1 vs. 5637 | -0.5740 | -0.6379 to -0.5101 | Yes |
| SV-HUC-1 vs. T24 | -0.5420 | -0.6059 to -0.4781 | Yes |
| RT4 vs. BIU-87 | -0.008000 | -0.07190 to 0.05590 | No |
| RT4 vs. 5637 | -0.4300 | -0.4939 to -0.3661 | Yes |
| RT4 vs. T24 | -0.3980 | -0.4619 to -0.3341 | Yes |
| BIU-87 vs. 5637 | -0.4220 | -0.4859 to -0.3581 | Yes |
| BIU-87 vs. T24 | -0.3900 | -0.4539 to -0.3261 | Yes |
| 5637 vs. T24 | 0.03200 | -0.03190 to 0.09590 | No |
|  |  |  |  |

| Tukey's multiple comparisons test | Mean Diff. | 95% CI of diff. | Significant |
| --- | --- | --- | --- |
|  |  |  |  |
| SV-HUC-1 vs. RT4 | -0.2860 | -0.4321 to -0.1399 | Yes |
| SV-HUC-1 vs. BIU-87 | -0.3220 | -0.4681 to -0.1759 | Yes |
| SV-HUC-1 vs. 5637 | -0.8440 | -0.9901 to -0.6979 | Yes |
| SV-HUC-1 vs. T24 | -0.8660 | -1.012 to -0.7199 | Yes |
| RT4 vs. BIU-87 | -0.03600 | -0.1821 to 0.1101 | No |
| RT4 vs. 5637 | -0.5580 | -0.7041 to -0.4119 | Yes |
| RT4 vs. T24 | -0.5800 | -0.7261 to -0.4339 | Yes |
| BIU-87 vs. 5637 | -0.5220 | -0.6681 to -0.3759 | Yes |
| BIU-87 vs. T24 | -0.5440 | -0.6901 to -0.3979 | Yes |
| 5637 vs. T24 | -0.0220 | -0.1681 to 0.1241 | No |
|  |  |  |  |
